# MBD3 expression and DNA binding patterns are altered in a rat model of temporal lobe epilepsy

**DOI:** 10.1038/srep33736

**Published:** 2016-09-21

**Authors:** Joanna Bednarczyk, Konrad J. Dębski, Anna M. Bot, Katarzyna Lukasiuk

**Affiliations:** 1 Laboratory of Epileptogenesis, Department of Molecular and Cellular Neurobiology, Nencki Institute of Experimental Biology of Polish Academy of Sciences, Warsaw, Poland; 2 Laboratory of Bioinformatics, Neurobiology Center, Nencki Institute of Experimental Biology of Polish Academy of Sciences, Warsaw, Poland

## Abstract

The aim of the present study was to examine involvement of MBD3 (methyl-CpG-binding domain protein 3), a protein involved in reading DNA methylation patterns, in epileptogenesis and epilepsy. We used a well-characterized rat model of temporal lobe epilepsy that is triggered by status epilepticus, evoked by electrical stimulation of the amygdala. Stimulated and sham-operated animals were sacrificed 14 days after stimulation. We found that MBD3 transcript was present in neurons, oligodendrocytes, and astrocytes in both control and epileptic animals. We detected the nuclear localization of MBD3 protein in neurons, mature oligodendrocytes, and a subpopulation of astrocytes but not in microglia. Amygdala stimulation significantly increased the level of MBD3 immunofluorescence. Immunoprecipitation followed by mass spectrometry and Western blot revealed that MBD3 in the adult brain assembles the NuRD complex, which also contains MTA2, HDAC2, and GATAD2B. Using chromatin immunoprecipitation combined with deep sequencing, we observed differences in the occupancy of DNA regions by MBD3 protein between control and stimulated animals. This was not followed by subsequent changes in the mRNA expression levels of selected MBD3 targets. Our data demonstrate for the first time alterations in the MBD3 expression and DNA occupancy in the experimental model of epilepsy.

Epilepsy is one of the most common neurological disorders, affecting approximately 1% of the population^1^. In about 30% of cases, epilepsy results from an insult in the brain, such as traumatic brain injury, stroke, brain infection, or status epilepticus (SE)^2^. In such cases, the initial insult is followed by a latency period (epileptogenesis) before the appearance of spontaneous seizures and diagnosis of epilepsy^3^. During epileptogenesis and epilepsy, several molecular and cellular changes occur, including alterations in gene and protein expression^4^,^5^. The mechanisms that govern alterations in gene expression in epileptogenesis and epilepsy are not well understood. Recently, epigenetic mechanisms, including DNA methylation, have emerged as potentially important in the transcription regulation of these processes^6^,^7^. Experimental evidence supports this possibility, including increases in the levels of DNA methylases and increases in global DNA methylation in the hippocampus in temporal lobe epilepsy (TLE) patients and animals with experimental epilepsy^8^,^9^,^10^,^11^. Alterations in global methylation status were also observed in the hippocampus in epileptic rats and humans with TLE^11^,^12^. The regulatory involvement of promoter methylation in the expression of several epilepsy- or seizure-related genes, including reelin, *Grin2b/Nr2b* (glutamate receptor, ionotropic, *N*-methyl-*D*-aspartate 2b), *Gria2* (glutamate receptor, ionotropic, AMPA 2), and *Bdnf* (brain-derived neurotrophic factor), has been suggested^8^,^13^,^14^.

In this context, increasing evidence indicates the importance of DNA methylation, suggesting a role for molecular machinery that is involved in reading DNA methylation patterns in epilepsy. Interesting candidate proteins belong to an evolutionarily conserved family of MBD proteins that are characterized by the presence of a methyl-CpG binding domain (MBD) and are believed to read DNA methylation patterns^15^. Interestingly, mutations in methyl CpG binding protein 2 (MeCP2), a member of the MBD protein family, have been associated with Rett syndrome, which is characterized by severe epilepsy^16^,^17^.

Our previous microarray studies revealed that the expression level of methyl-CpG binding domain 3 (*Mbd3*) gene, which encodes MBD3 protein that belongs to the MBD protein family, is increased in the temporal lobe in a rat model of TLE^18^. MBD3 is unique among MBD protein family members because of a mutation in the MBD domain that causes loss of the ability to selectively recognize methyl-CpGs and because it can bind to 5-hydroxymethylcytosine and unmethylated DNA^15^. MBD3 has been suggested to influence gene expression as a component of the multisubunit nucleosome remodeling and deacetylation (NuRD) complex, and it is indispensable for the assembly of this complex^15^. The NuRD complex comprises subunits with enzymatic activity, including histone deacetylase (HDAC1/2) and chromodomain helicase DNA-binding protein (CHD3/4), and other components like metastasis-associated protein (MTA1/2/3), retinoblastoma-binding protein (RBBP7/4), and GATA zinc finger domain containing (GATAD2A/2B) that are crucial for the action of the NuRD complex in the epigenetic regulation of gene expression. The role of the MBD3/NuRD complex in brain function is based on its involvement in the choice of the appropriate cell lineage and differentiation of the rodent cerebellar cortex and cerebral cortex *in vivo*^19^,^20^ and the existence of a single-nucleotide polymorphism (SNP) variant that was identified in autistic individuals^21^. However, no data are available on the involvement of MBD3/NuRD in epilepsy.

The aim of the present study was to examine the localization of MBD3 mRNA and protein in temporal lobe structures in control and epileptic animals. We evaluated differences in binding of the MBD3/NuRD complex to DNA regions between control and epileptic brains and how such binding affects the mRNA expression of select target genes. Such data will provide useful information for understanding the events occurring in epileptogenesis and epilepsy.

## Results

### *Mbd3* gene is expressed in a cell-specific manner in the normal and epileptic brain

**Figure 1:**
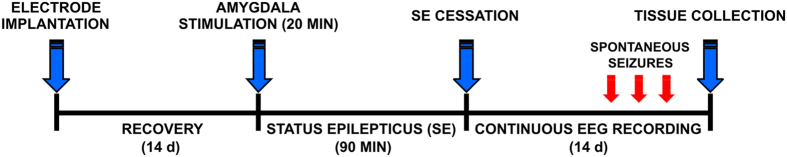
Experimental design. Animals underwent electrode implantation and after 14 d recovery status epilepticus was evoked by amygdala stimulation. Status epilepticus was stopped 90 min after stimulation by injection of diazepam. The rats were continuously monitored by video electroencephalography to detect spontaneous epileptic seizures until the end of experiment. Tissue for subsequent analyses was collected 14 d after stimulation.

**Figure 2:**
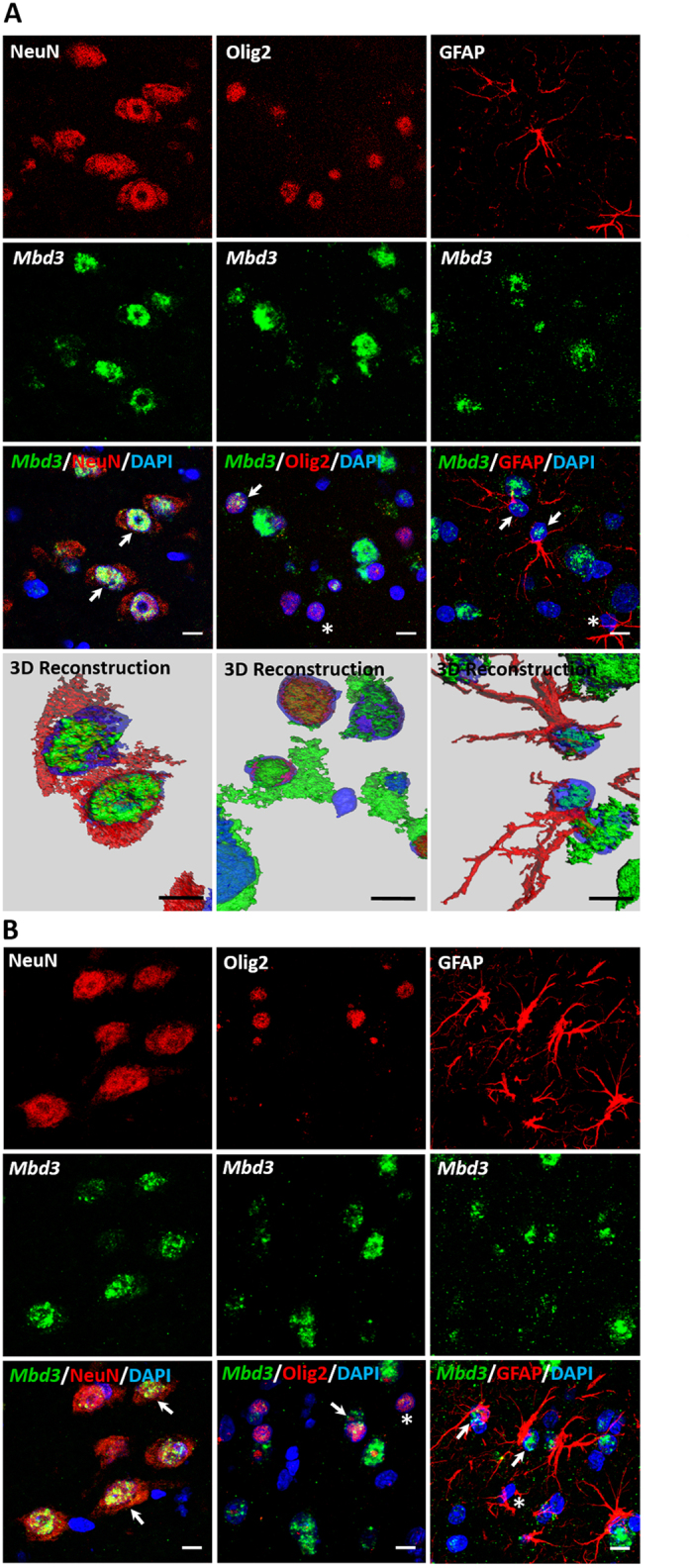
Expression pattern of *Mbd3* mRNA in normal and stimulated rat brain. Cellular localization of *Mbd3* transcript in the piriform cortex in sham-operated rats (**A**) and amygdala-stimulated rats 14 days after status epilepticus (**B**). Representative confocal microscopy images show *Mbd3* mRNA (green), detected by *in situ* hybridization, and neuronal cells, oligodendrocytes, and astrocytes (red), detected by immunohistochemistry using NeuN, Olig2, and GFAP antibodies, respectively. Cell nuclei were counterstained with DAPI (blue). Typical examples of triple-labeled cells are indicated by arrows. Olig2- and GFAP-positive cells that lack *Mbd3* mRNA are marked with asterisks. DAPI. 4,6-diamidino-2-phenylindole; GFAP, glial fibrillary acidic protein; NeuN, neuron-specific nuclear protein; Olig2, oligodendrocyte lineage transcription factor 2. Scale bar = 10 μm.

The design of the study is summarized on Fig. 1. To investigate the expression and localization of *Mbd3* mRNA in the brain in Sprague-Dawley rats, we performed *in situ* hybridization combined with immunofluorescence staining using a probe for *Mbd3* and antibodies that specifically recognize particular cell types. Confocal microscopic studies revealed that the *Mbd3* transcript is widely expressed in the normal brain and is present in neurons, oligodendrocytes, and astrocytes (Fig. 2A). Interestingly, we found that *Mbd3* mRNA is present only in a fraction of oligodendrocytes and astrocytes (Fig. 2A,B, arrow), whereas other oligodendrocytes and astrocytes are devoid of *Mbd3* mRNA (Fig. 2A,B, asterisk). We next examined the effect of epileptogenic insult (i.e., SE evoked by amygdala stimulation) on the pattern of expression and cell-specific localization of *Mbd3* mRNA using a rat model of TLE. Fourteen days following amygdala stimulation, we observed the same pattern for *Mbd3* mRNA expression as in the control brain (Fig. 2B).

**Figure 3:**
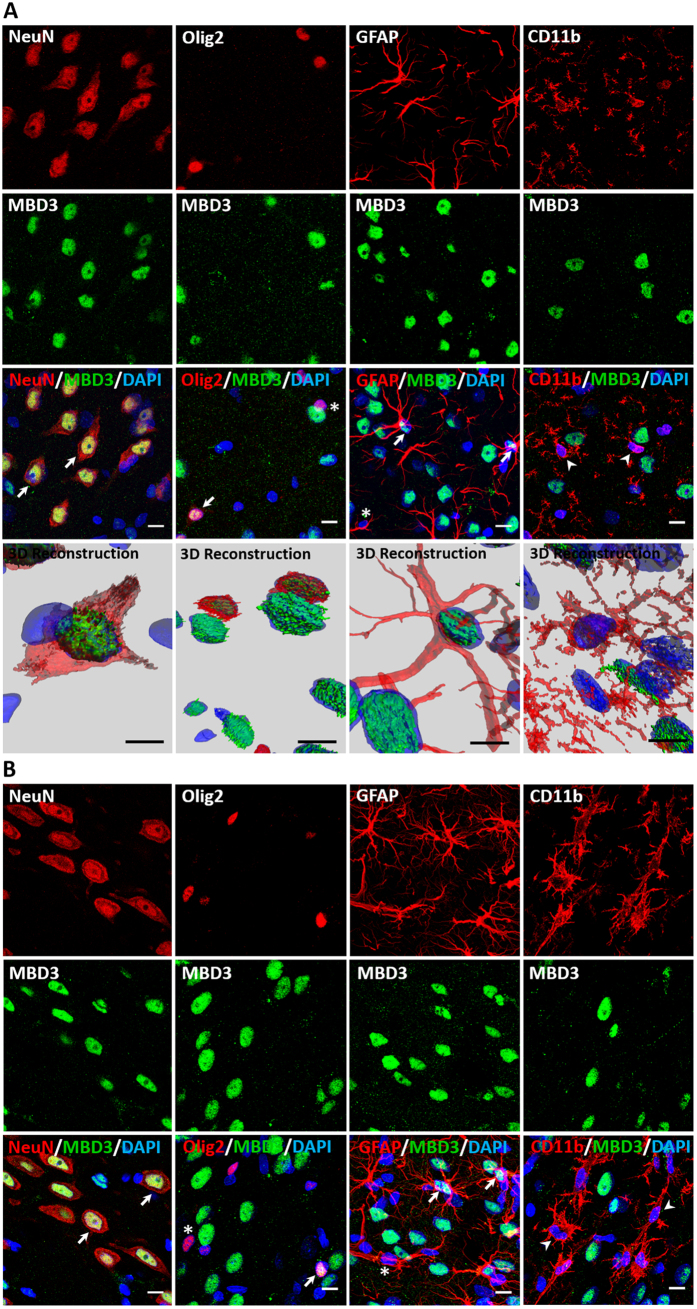
Cellular localization of MBD3 protein in normal and epileptic rat brain. MBD3 immunofluorescence in different cell types in the piriform cortex in sham-operated rats (**A**) and epileptic rats 14 days after amygdala stimulation (**B**). Representative confocal microscopy images show MBD3 immunostaining (green) and neuronal cells, oligodendrocytes, astrocytes, and microglia (red), detected by immunohistochemistry using NeuN, Olig2, GFAP, and CD11b antibodies, respectively. Cell nuclei were counterstained with DAPI (blue). Typical examples of triple-labeled cells are indicated by arrows. Olig2- and GFAP-positive cells that lack MBD3 protein are marked with asterisks. Examples of microglial cells that do not express MBD3 protein are indicated by arrowheads. CD11b, cluster of differentiation molecule 11B, integrin αM chain; DAPI, 4,6-diamidino-2-phenylindole; GFAP, glial fibrillary acidic protein; MBD3, methyl-CpG binding domain protein 3; NeuN, neuron-specific nuclear protein; Olig2, oligodendrocyte lineage transcription factor 2. Scale bar = 10 μm.

We next characterized the expression profile of MBD3 protein in sham-operated and epileptic animals. The expression pattern of MBD3 protein was similar to that for *Mbd3* transcript. The expression of MBD3 protein was widespread and abundant. MBD3 immunoreactivity was present in the nuclei of neurons, oligodendrocytes, and astrocytes but not in microglia in both normal and epileptic animals (Fig. 3A,B). MBD3 was expressed in almost every neuron, whereas we did not find MBD3 protein in some glial fibrillary acidic protein (GFAP)-positive and Olig2-positive cells (Fig. 3A,B, asterisk).

To assess the percentage of neurons that contained MBD3 protein, we counted the number of double-labeled NeuN- and MBD3-positive cells in the piriform cortex, entorhinal cortex, and central, lateral, basal, basomedial, and medial nuclei of the amygdala. Quantitative analyses revealed that MBD3 protein was expressed in the control brain ipsilaterally in 98.0 ± 1.5% to 99.7 ± 0.5% of neurons and contralaterally in 99.8 ± 2.0% to 99.9 ± 2.3% of neurons, depending on the structure (Supplementary Table 1). Fourteen days after amygdala stimulation, the proportion of neurons that expressed MBD3 was 96.8 ± 0.1% to 99.6 ± 0.1% ipsilaterally and 99.4 ± 1.1% to 99.9 ± 0.2% contralaterally, depending on the brain region (Supplementary Table 1).

**Figure 4:**
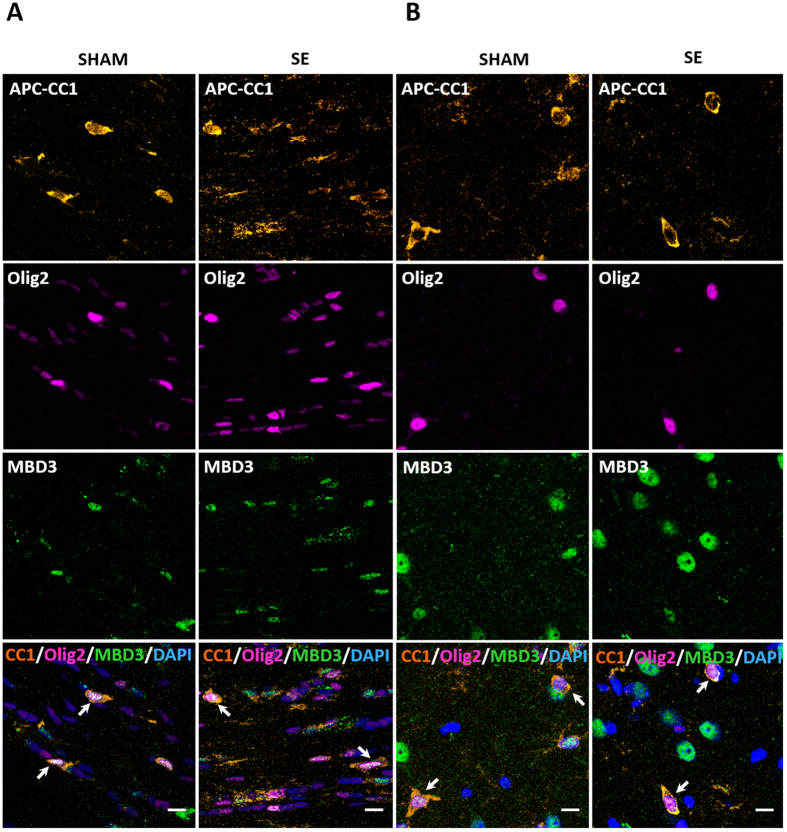
Characterization of oligodendrocytes that express MBD3 protein in the brain. MBD3 protein in oligodendrocytes in white matter in the corpus callosum (**A**) and piriform cortex (**B**). Representative confocal microscopy images of both normal and amygdala-stimulated rats show MBD3 immunoreactivity (green), Olig2-positive oligodendrocytes (magenta), and APC-CCI-positive mature oligodendrocytes (orange). Cell nuclei were counterstained with DAPI (blue). Typical examples of mature oligodendrocytes that express MBD3 are indicated by arrows. APC-CC1, adenomatous polyposis coli; DAPI, 4,6-diamidino-2-phenylindole; MBD3, methyl-CpG binding domain protein 3; Olig2, oligodendrocyte lineage transcription factor 2. Scale bar = 10 μm.

We found that only a fraction of oligodendrocytes contained MBD3, and we next characterized the oligodendrocyte population by immunostaining with APC-CC1, a marker of mature oligodendrocytes. We found that MBD3 protein was expressed in mature oligodendrocytes in the corpus callosum (i.e., the main reservoir of differentiated oligodendrocytes) in both sham-operated and epileptic rats (Fig. 4A). Moreover, mature oligodendrocytes that expressed MBD3 were also observed in gray matter (Fig. 4B).

### MBD3 immunoreactivity is increased following amygdala stimulation

**Figure 5:**
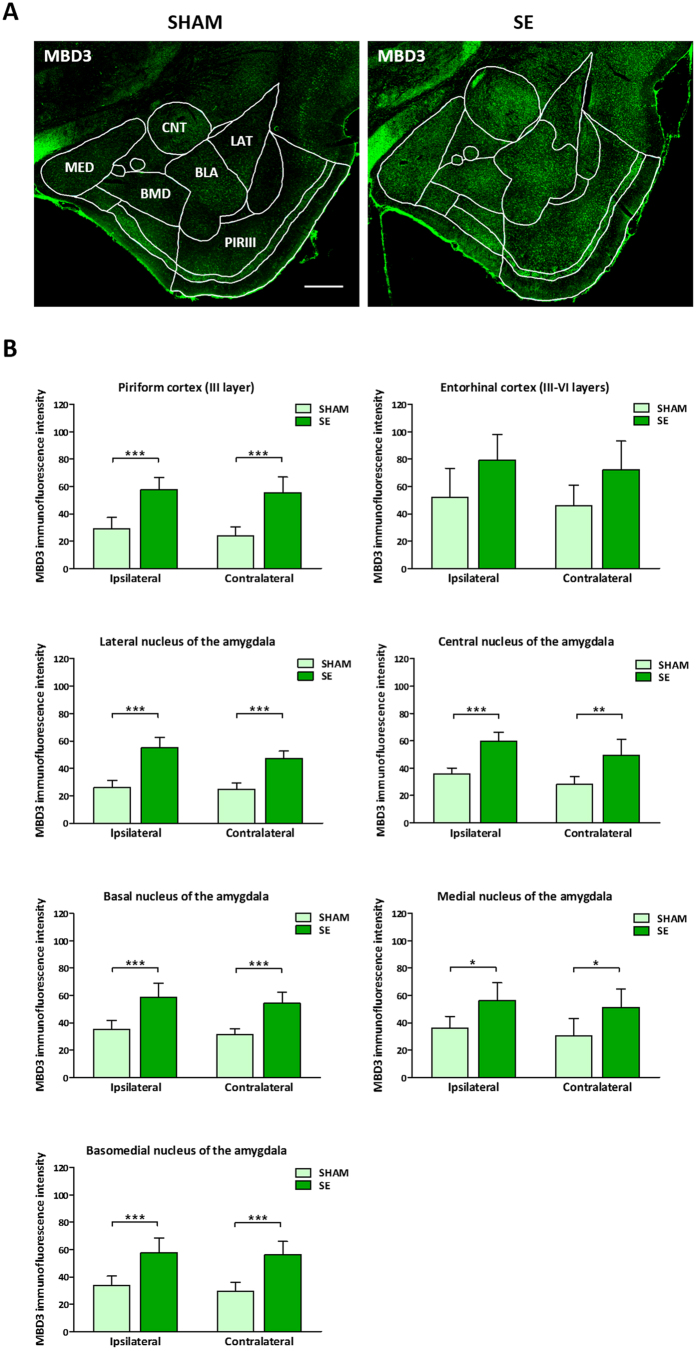
Level of MBD3 immunofluorescence in normal vs. epileptic animals. (**A**) Representative images of temporal lobe structures in sham-operated and epileptic rats, immunostained with MBD3 antibody (green). (**B**) Quantitative analysis of immunofluorescence levels in selected structures in control and epileptic animals. The data are expressed as the relative level of MBD3 immunoreactivity ± SD. *n* = 5. *P < 0.05, **P < 0.01, ***P < 0.001 (two-way ANOVA). BLA, basolateral nucleus of the amygdala; BMD, basomedial nucleus of the amygdala; CNT, central nucleus of the amygdala; LAT, lateral nucleus of the amygdala; MBD3, methyl-CpG binding domain protein 3; MED, medial nucleus of the amygdala; PIRIII, piriform cortex. Scale bar = 500 μm.

Amygdala stimulation-evoked SE induced a clear increase in the level of MBD3 immunofluorescence 14 days after stimulation (Fig. 5A). Quantitative analyses revealed an increase in MBD3 immunofluorescence in layer III of the piriform cortex and amygdala in epileptic rats compared with controls (Fig. 5B). The most prominent differences in the intensity of MBD3 immunoreactivity between groups were detected in the ipsi- and contralateral third layer of the piriform cortex (107.7 ± 17.9% and 132.1 ± 23.5%, respectively; *P* < 0.001). We observed 110.5 ± 20.2% and 90.1 ± 17.5% (*P* < 0.001) increases in MBD3 immunofluorescence in the ipsilateral and contralateral lateral nucleus of the amygdala, respectively. We detected 66.3 ± 13.9% (*P* < 0.001) and 75.8 ± 17.2% (*P* < 0.01) increases in the levels of MBD3 protein in the ipsilateral and contralateral central nucleus of the amygdala in epileptic animals, respectively. In the ipsi- and contralateral basal nucleus of the amygdala, we observed 66.8 ± 15.8% and 72.8 ± 15.6% (*P* < 0.001) increases in MBD3 immunofluorescence following epileptogenic insult, respectively. Furthermore, we observed 71.5 ± 16.7% and 90.4 ± 18.6% higher levels of MBD3 immunoreactivity in epileptic animals than in control animals in the ipsilateral and contralateral basomedial nucleus of the amygdala, respectively (*P* < 0.001). A less pronounced increase in the intensity of MBD3 immunofluorescence was observed in the ipsilateral and contralateral medial nucleus of the amygdala, where we observed 55.9 ± 15.9% and 68.0 ± 20.0% increases in stimulated animals compared with controls, respectively (*P* < 0.05). The differences in MBD3 immunoreactivity in the entorhinal cortex between groups did not reach statistical significance.

### The MBD3/NuRD complex assembles in the adult rat brain

**Figure 6:**
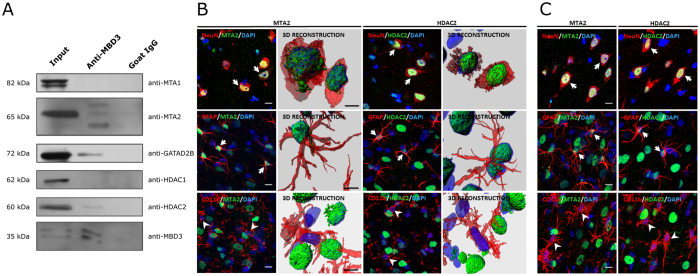
Composition of MBD3-containing complex and cellular localization of NuRD subunits in the brain. (**A**) Representative immunoblotting with antibodies directed against MTA1, MTA2, HDAC1, HDAC2, and GATAD2B proteins in lysates of temporal lobe structures subjected to immunoprecipitation with MBD3 antibody. (**B**) Cellular localization of MTA2 and HDAC2 proteins in the piriform cortex in sham-operated animals. (**C**) Cellular localization of MTA2 and HDAC2 proteins in the piriform cortex in epileptic rats 14 days after amygdala stimulation. (**B,C**) Representative confocal microscopy images show MTA2 and HDAC2 proteins (green) and neuronal cells, astrocytes, and microglia (red), detected by immunohistochemistry using NeuN, GFAP, and CD11b antibodies, respectively. Cell nuclei were counterstained with DAPI (blue). Typical examples of triple-labeled cells are indicated by arrows. Examples of microglial cells that do not express MTA2 and HDAC2 proteins are indicated by arrowheads. CD11b, cluster of differentiation molecule 11B, integrin αM chain; DAPI, 4,6-diamidino-2-phenylindole; GATAD2B, GATA zinc finger domain containing 2B; GFAP, glial fibrillary acidic protein; HDAC2, histone deacetylase 2; MBD3, methyl-CpG binding domain protein 3; MTA1, metastasis associated 1 family, member 1; MTA2, metastasis associated 1 family, member 2; NeuN, neuron-specific nuclear protein;. Scale bars = 10 μm. Full length scans of Western Blots used for images cropped to the Fig. 6 are presented in Supplementary Figure 1.

To verify that MBD3 is a member of the NuRD complex in the brain, we first purified the MBD3-containing complex by immunoprecipitation with an antibody directed against MBD3 and used mass spectrometry to identify other components of the complex. Mass spectrometry revealed the following components of the NuRD complex in the immunoprecipitate: HDAC1/2, MBD3, RBBP4, MTA1/2, and GATAD2B (Table 1). This was also confirmed by the coimmunoprecipitation analyses followed by Western blot, in which endogenous MBD3 formed a complex with HDAC2, MTA2 and GATAD2B in rat temporal lobe structures (Fig. 6A). Western blot did not confirm presence of RBBP4 protein in the MBD3 containing complex (not shown). These results indicate that an MBD3-containing NuRD complex is assembled in the adult rat brain.

We next characterized the cellular localization of MTA2 and HDAC2 proteins (i.e., components of the MBD3 containing NuRD complex in the brain). Double-immunohistochemistry revealed that MTA2 and HDAC2 proteins were expressed in neurons and astrocytes in sham-operated animals (Fig. 6B). Interestingly, we did not find MTA2 or HDAC2 proteins in microglia. The same MTA2 and HDAC2 expression patterns were observed in epileptic brains (Fig. 6C). No differences in MTA2 and HDAC2 localization were found between control and epileptic animals. Altogether, these data suggest that the NuRD complex is present in the brain in a cell-specific manner and assembled in neurons and a fraction of oligodendrocytes and astrocytes but not in microglia.

### The MBD3/NuRD complex binds to different DNA regions in control and amygdala-stimulated animals

Because of differences in the expression levels of MBD3 protein between normal and amygdala-stimulated rats, we hypothesized that the MBD3-containing NuRD complex targets different DNA regions in control *vs*. stimulated animals. To detect DNA regions that are occupied by the MBD3-containing complex, we performed ChIP-Seq analyses using blocks of tissue that contained the entorhinal, piriform, and perirhinal cortices, the piriform nucleus, and the amygdala.

**Figure 7:**
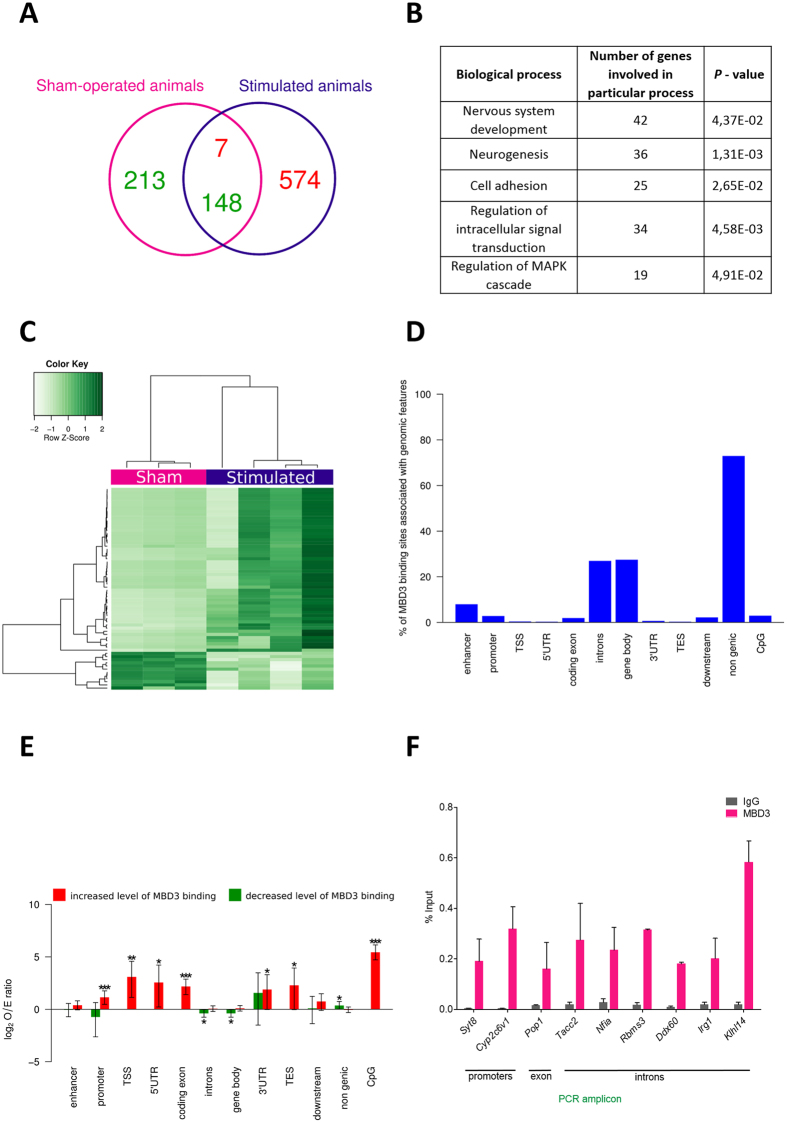
MBD3 DNA binding in control and amygdala-stimulated rats. (**A**) Venn diagram showing numbers of DNA regions occupied by MBD3 in sham-operated (magenta) and stimulated (dark blue) rats. Regions with increases and decreases in MBD3 occupancy in stimulated animals are indicated in red and green, respectively. (**B**) Functional annotation of genes associated with sites that were differentially occupied by MBD3 in control and stimulated animals. (**C**) Heatmap of DNA regions that were differentially occupied by MBD3 in control and stimulated animals (FDR < 0.1). Each column represents an individual animal, and each row represents the genome region. Colors on the heatmap represent the Z-score, with relatively high (dark green) and relatively low (light green) levels of MBD3 binding to DNA. (**D**) Distribution of sites differentially (cut-off *P* < 0.01) occupied by MBD3 in stimulated animals in defined genomic features (TSS, TES, gene body, exon, intron, UTR, downstream, enhancer, promoter, CpG, and non-genic). Notice that a single peak can overlap with more than one type of feature in this analysis. (**E**) Frequency of observed MBD3 binding changes (cut-off *P* < 0.01) in stimulated animals compared with sites with no changes in MBD3 binding, with upper and lower 95% confidence intervals for defined genomic features. Increased binding of MBD3 relative to controls is coded with red bars, and decreased binding of MBD3 relative to controls is coded with green bars. O/E, observed/expected ratio. *P < 0.05, **P < 0.01, ***P < 0.001 (Fisher’s Exact test). (**F**) qPCR validation of MBD3 binding at nine selected target loci in sham-operated rats. Average C_t_ values for individual ChIP and IgG controls are expressed as a percentage of input ± SD.

We identified regions of MBD3 binding by comparing precipitations from temporal lobe structures with input samples. We used MACS2 software followed by the DiffBind R/Bioconductor package. Using this approach, we were able to detect 942 binding sites that were differentially occupied by MBD3 protein in normal and stimulated animals (*P* < 0.01; Fig. 7A). MBD3 binding was found for 213 and 574 DNA regions exclusively in control and amygdala-stimulated rats, respectively. Aside from the differences in the number of binding sites between groups, we identified 155 regions that were occupied by MBD3 in both control and amygdala-stimulated animals. For seven regions, we detected enrichment of MBD3 occupancy (Fig. 7A, red), and for 148 regions, we detected depletion of MBD3 occupancy following SE (Fig. 7A, green) relative to controls. The gene ontology analysis demonstrated that the genes that were nearest to the MBD3 peaks are involved in various processes, including nervous system development, neurogenesis, and the regulation of intracellular signal transduction (Fig. 7B). Hierarchical clustering revealed that sham-operated controls differed from stimulated animals (Fig. 7C). The heatmap with unsupervised clustering distinguished two groups of DNA regions that were differentially occupied by MBD3. The first contained regions that were characterized by a relatively high frequency of MBD3 occupancy (Fig. 7C, dark green), and the second one had a relatively low frequency of MBD3 association (Fig. 7C, light green) in amygdala-stimulated animals.

The distribution of sites that were differentially occupied by MBD3 in different genomic features revealed that a high proportion of MBD3 binding occurred in non-genic regions (72.9%; Fig. 7D). Furthermore, many of the sites that were differentially occupied by MBD3 were assigned to gene bodies, particularly introns. In contrast, only a few sites that were differentially occupied by MBD3 were observed in transcriptional start and end sites, UTRs (untranslated region), and exon sequences.

The analysis of the frequency of changes in MBD3 binding revealed that promoter regions, transcriptional start and end sites, UTRs, the coding part of exons, and CpG islands were more frequently occupied by MBD3 in stimulated animals compared with non-differentially occupied binding sites (Fig. 7E). In sham-operated rats, a higher than expected frequency of MBD3 binding to non-genic regions was detected. For gene bodies and introns, the frequency of binding was lower than expected (Fig. 7E).

Binding to putative MBD3 target DNA regions was verified for nine selected genes by ChIP-qPCR. MBD3 binding was confirmed for all selected candidate regions (Fig. 7F). MBD3 binding was detected in promoters of *Syt8* (synaptotagmin 8; calcium-induced membrane fusion during vesicular traffic) and *Cyp2c6v* (cytochrome P450, family 2, subfamily C, polypeptide 6), exons of *Pop1* (processing of precursor 1, ribonuclease P/MRP subunit; pre-RNA processing), and introns of *Tacc2* (transforming, acidic coiled-coil containing protein 2; centrosome- and microtubule-interacting protein), *Nfia* (nuclear factor I/A; transcription factor)*, Rbms3* (RNA binding motif, single-stranded interacting protein 3; tumor suppressor gene, RNA-binding protein)*, Ddx60* (DEAD [Asp-Glu-Ala-Asp] box polypeptide 60; RNA helicase)*, Irg1* (immunoresponsive 1 homolog; negative regulator of Toll-like receptors), and *Klhl14* (Kelch-like family member 14) (Fig. 7F). The positive verification of MBD3 binding to all of the evaluated DNA regions indicated that the ChIP-Seq analysis was sufficiently stringent to avoid substantial false-positive findings.

### mRNA expression levels of selected genes with altered MBD3 binding status in control *vs*. stimulated animals

**Figure 8:**
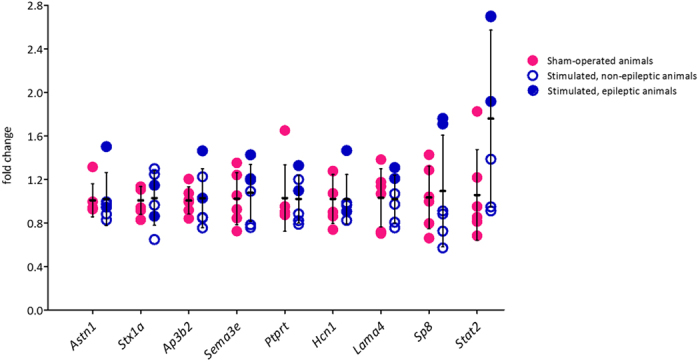
mRNA expression levels for selected genes with altered MBD3 binding status in control vs. stimulated rats. mRNA expression in the temporal lobe in sham-operated (magenta) and stimulated (dark blue) rats was evaluated with qRT-PCR. The data were normalized to *Hprt1* expression levels and are expressed as fold changes in expression ± SD. *n* = 6. The data were analyzed by two-tailed *t*-test.

To assess the possible influence of the MBD3-containing complex on the expression level of target genes, we used qPCR to evaluate the mRNA expression levels of nine genes that are relevant to seizures or epilepsy: *Astn1* (astrotactin 1), *Stxa1* (syntaxin 1A [brain]), *Ap3b2* (adaptor-related protein complex 3, β2 subunit), *Sema3e* (sema domain, Ig domain, short basic domain, secreted, [semaphorin] 3E), *Lama4* (laminin, α4), *Sp8* (Sp8 transcription factor), *Ptprt* (protein tyrosine phosphatase, receptor type, T), *Hcn1 (Hcn1*, hyperpolarization activated cyclic nucleotide gated potassium channel 1), and *Stat2* (signal transducer and activator of transcription 2). We did not find any significant differences in the expression levels of MBD3 target genes between control and amygdala-stimulated rats. Interestingly, the expression level of Sp8 and Stat2 mRNA in epileptic amygdala-stimulated animals was higher than in sham-operated and other stimulated rats (Fig. 8).

## Discussion

The present study was designed to evaluate whether MBD3 protein is involved in temporal lobe epilepsy pathology in an experimental rat model. We found that *Mbd3* mRNA and MBD3 protein were expressed in a cell-specific manner in control and diseased brains and that MBD3 expression increased in the piriform cortex and amygdala following the epileptogenic stimulus. Moreover, we observed alterations in the DNA binding of the MBD3-containing complex in experimental animals compared with controls, indicating the involvement of MBD3 in chromatin function and/or the regulation of gene expression in epileptogenesis and epilepsy.

We employed a well-characterized model of TLE in rats, in which epilepsy is triggered by SE that is evoked by amygdala stimulation^22^,^23^. Following the cessation of SE, rats are seizure-free for up to several weeks and then begin to express spontaneous seizures. Neurodegeneration, gliosis, and alterations in gene and protein expression in temporal lobe structures in this model have been extensively characterized^18^,^22^,^23^,^24^. *Mbd3* is an interesting gene that is upregulated in temporal lobe structures 14 days after stimulation in this model, as detected by microarray analysis^18^.

Previous studies have reported the neuronal expression of *Mbd3* mRNA and MBD3 protein^25^,^26^. Our study extends this observation by showing that MBD3 protein is present in almost all neurons. The expression of MBD3 in MAP2-negative cells *in vitro* has also been reported, but these cells have not been further characterized^25^. We found that *Mbd3* gene and MBD3 protein are expressed in a fraction of oligodendrocytes and astrocytes but not in microglia. This pattern of *Mbd3* mRNA and MBD3 protein expression was observed in both normal and epileptic rat brains.

An interesting finding in the present study was the lack of MBD3 expression in microglia and a fraction of oligodendrocytes and astrocytes. This implies that microglia and subpopulations of oligodendrocytes and astrocytes employ different epigenetic mechanisms than other cells and do not utilize the MBD3-containing NuRD complex to read DNA cytosine methylation status. The difference in the composition of DNA methylation-detecting complexes in different cell types is also supported by the cellular localization of MTA2 and HDAC2 proteins, which are also components of the MBD3-containing NuRD complex, in the brain. We detected the expression of MTA2 and HDAC2 in neurons, which is consistent with previous studies^27^,^28^,^29^,^30^. We also observed the expression of both HDAC2 and MTA2 in astrocytes. The level of HDAC2 expression in astrocytes was clearly lower than in neuronal cells. These results are consistent with a recent report that showed weaker immunofluorescence staining of HDAC2 protein in astroglia compared with neurons in the mouse cerebellum 5 days after birth^30^. Interestingly, in previous studies, HDAC2 was not detected in astrocytes in the adult hippocampus, indicating that HDAC2-positive astrocytes are not present in all brain structures^28^,^29^. We did not observe the expression of HDAC2 or MTA2 in microglia. Considering the complex pattern of expression of components of the NuRD complex in different cell types and subpopulations, we suggest that the composition of the MBD3/NuRD complex is different in various types of cells in the brain.

The fact that MBD3 is present only in some oligodendrocytes suggests that it marks distinct functional classes of those glial cells. Indeed, our data indicate that MBD3 is expressed in mature oligodendrocytes. This contrasts with another protein that belongs to the MBD family, MeCP2, which is expressed in both mature oligodendrocytes and oligodendrocyte progenitor cells^31^,^32^. The lack of MBD3 in a fraction of oligodendrocytes indicates that this protein, in contrast to MeCP2, is not essential for the proper functions of at least some oligodendrocytes^32^. Interestingly, mice that lost MeCP2 specifically in oligodendrocyte lineage cells developed abnormal behavioral symptoms that were characteristic of Rett syndrome, and the levels of some myelin-related proteins were impaired in these mice. Similar to oligodendrocytes, we found that only a select population of GFAP-positive cells expressed MBD3 protein in astrocytes. Interestingly, the expression of MeCP2 in astrocytes is essential for the regulation of BDNF function, cytokine production, and proper dendritogenesis^31^,^33^. Remaining to be determined is whether the dysfunction of MBD3 in a fraction of oligodendrocytes or astrocytes leads to such severe phenotypes as in the case of MeCP2.

Although the MBD3/NuRD complex has been studied *in vitro* in embryonic stem cells and *in vivo* in developing rodent cerebral and cerebellar cortices^19^,^20^,^34^, the composition of the native MBD3/NuRD complex in the adult rat brain has remained unexplored. Our immunoprecipitation-mass spectrometry data followed by Western blot verification indicated that MBD3 formed a complex with HDAC2, MTA2, and GATAD2B in temporal lobe structures and hence may participate in the epigenetic regulation of gene expression in neurons and subpopulations of oligodendrocytes and astrocytes as a component of the NuRD complex.

Our study revealed that the level of MBD3 immunoreactivity in neurons in epileptic brains was significantly higher than in sham-operated controls. To our knowledge, this is the first report on alterations in the levels of MBD3 protein in brain pathology. Previously, only changes in mRNA levels were studied. Importantly, Francis *et al.* reported an increase in *Mbd3* mRNA in a kindling model of epileptogenesis^35^. This finding is consistent with the notion that an increase in MBD3 expression is involved in epilepsy pathology. Interestingly, a decrease in *Mbd3* mRNA expression was observed following transient forebrain ischemia^36^. Considering the neuronal localization of MBD3, we posit this was attributable to massive ischemia-induced neurodegeneration. We propose that an increase in MBD3 protein levels results in higher levels of the MBD3/NuRD complex in temporal lobe structures in rats that are subjected to epileptogenic insult, which may play a pivotal role in the epigenetic regulation of processes that occur during epileptogenesis in the brain.

To further explore the possible involvement of the MBD3-containing NuRD complex in epigenetic events in the epileptic brain, we characterized DNA regions that were occupied by the MBD3-containing complex in control *vs*. stimulated brains. We found differences in the frequency of MBD3/NuRD binding to the same DNA regions between normal and amygdala-stimulated animals. Our data revealed a large number of MBD3/NuRD binding sites throughout the genome, which were localized to promoters, exons, introns, and intergenic regions in the brain. Such a distribution of MBD3 binding to genomic features is consistent with previous studies that reported abundant occupancy of the complex at promoters, gene bodies, and enhancers of active genes *in vitro*^37^,^38^.

Interestingly, several genes that were targeted by MBD3/NuRD in the present study have been linked to epilepsy. Examples are *Stx1a* and *Ptprt* (which encodes syntaxin-binding protein). *De novo* mutations of syntaxin-binding protein 1 were found in patients with Ohtahara syndrome, an early infantile epileptic encephalopathy^39^. The attenuated binding of a mutant form of syntaxin-binding protein 1 to syntaxin 1 inhibited the formation of the SNARE (soluble *N*-ethylmaleimide-sensitive factor attachment protein receptor) complex, resulting in severe epilepsy^40^. Reductions of *Hcn1* mRNA levels have been described in the hippocampus after kindling and kainic acid-induced SE in adult rats, supporting the involvement of *Hcn1* in epileptogenesis^41^. *Stat2* has been proposed to contribute to seizure susceptibility^42^. Our data, however, do not support the notion that alterations in MBD3 binding are sufficient to influence the level of expression of these genes. We did not detect changes in mRNA levels in any of the nine investigated genes with altered MBD3 binding status in stimulated animals. Interestingly, *Sp8* and *Stat2* mRNA levels in some stimulated rats were higher than in sham-operated animals (Fig. 8). This suggests that some additional events (e.g., in the form of binding or activation of specific transcription factors) are needed for the effective regulation of gene expression, in addition to changes in MBD3 binding.

In conclusion, MBD3 localization was abundant in neurons, and the level of MBD3 protein increased following SE. The MBD3/NuRD complex assembles in the adult rat brain and is expressed in a cell-specific manner. MBD3/NuRD occupancy of the genome changed in amygdala-stimulated animals, indicating the involvement of MBD3 protein in epigenetic mechanisms that are triggered by epileptogenic insult, but alterations in MBD3 binding are not sufficient to drive changes at the level of gene expression. The relevance of changes in MBD3 expression and DNA occupancy for the pathological events undergoing in the brain during epileptogenesis and epilepsy is not clear. Further studies on the cooperation between the MBD3/NuRD complex and other DNA binding proteins during epileptogenesis are warranted to better understand the role of MBD3 in the development of epilepsy.

## Methods

### Amygdala stimulation-induced status epilepticus

All of the animal procedures were approved by the Ethical Committee on Animal Research of the Nencki Institute, and the experiments were conducted in accordance with the guidelines of Directive 2010/63/EU of the European Parliament and Council of the European Union (EU). Adult male Sprague-Dawley rats (290–320 g) were obtained from the Mossakowski Medical Research Centre (Polish Academy of Sciences, Warsaw, Poland) and housed in a controlled environment (24 °C, lights on 7:00 AM-7:00 PM) with free access to food and water. Status epilepticus was triggered by electrical stimulation of the amygdala as previously described^23^, with some modifications^22^. Stimulation and recording bipolar electrodes (Plastics One, Roanoke, VA, USA) were implanted in the left lateral nucleus of the amygdala under isoflurane anesthesia (2% in 100% oxygen). A surface recording electrode was implanted in the skull over the right frontal cortex. To evoke SE, the animals were electrically stimulated via the intra-amygdala electrode for 20 min with a 100-ms train of 1-ms biphasic square-wave pulses (400 μA peak to peak) delivered at 60 Hz every 0.5 s (Master-8 Stimulator connected to an ISO-Flex stimulus isolation unit; A.M.P.I., Israel). Status epilepticus was stopped 1.5 h after stimulation with an intraperitoneal injection of diazepam (20 mg/kg; Relanium, Polfa SA, Warsaw, Poland). The rats were continuously monitored by video electroencephalography to detect spontaneous epileptic seizures (Comet EEG, Grass Technologies, West Warwick, RI, USA). Sham-operated control animals had electrodes implanted but did not receive electrical stimulation. The experimental design is presented on Fig. 1.

### *In situ* hybridization

A complementary DNA fragment of MBD3 (nucleotides 483–1055; GenBank accession no. NM_001108735.1) was cloned into a pCRII-TOPO vector as previously described^26^. Using the linearized plasmids as a template, sense and antisense single-strand RNA probes were synthetized with a digoxigenin RNA labeling kit (11175025910, Roche Diagnostics, Basel, Switzerland). The rats were anesthetized with 70% carbon dioxide in air, followed by decapitation 14 days after stimulation. Brains were removed and frozen on pre-cooled heptane and stored at −80 °C. Coronal sections (10 μm) were cut, plated on poly-L-lysine-coated slides, and stored at −80 °C until processing. The sections were fixed with 4% paraformaldehyde in DEPC-phosphate-buffered saline (PBS; pH 7.4) for 10 min at room temperature, washed five times for 5 min each in DEPC-PBS, and then acetylated in a solution that contained 0.25% HCl, 0.25% acetic anhydride, and 0.1 M triethanolamine for 10 min. The sections were prehybridized for 2 h at 65 °C in a buffer that contained 5× SSC, 50% formamide, and 50 μg/ml salmon sperm DNA (pH 7.5). Following prehybridization, the sections were hybridized by incubating in hybridization buffer (H7782, Sigma, St. Louis, MO, USA) that contained probes for MBD3 (2.0 ng/μl) at 65 °C overnight and washed once for 30 min at 68 °C in 5× SSC and once for 20 min at 68 °C in 2× SSC, followed by incubation in 2× SSC enriched with RNase A (10 μg/ml) for 15 min at 37 °C. After washing twice for 10 min each at 37 °C in 0.2× SSC and three times for 10 min each in DEPC-PBS at room temperature, the sections were incubated in 1.0% H_2_O_2_ in DEPC-PBS for 15 min to inhibit endogenous peroxidase activity. They were then incubated for 1 h in blocking buffer (blocking reagent from TSA Kit, Molecular Probes, Eugene, OR, USA), followed by incubation for 1 h with anti-Digoxigenin-POD, Fab fragments (11633716001, Roche Diagnostics, Basel, Switzerland) diluted 1:500 in blocking buffer. The brain sections were then washed three times for 10 min each with DEPC-PBS and incubated for 20 min in AlexaFluor 488-conjugated tyramide (TSA Kit, T20948, Molecular Probes, Eugene, OR, USA) diluted 1:100 in 0.0015% H_2_O_2_/amplification buffer. The sections were then subjected to immunofluorescence staining as described below. As controls, hybridizations with a sense probe or omission of the probe were used. In both controls, the ISH signal was completely abolished. The results were confirmed in three independent brain sections from sham-operated and stimulated animals (*n* = 4). One of stimulated animals was epileptic, while remaining 3 animals did not experience spontaneous seizures (Table 2).

### Immunoprecipitation

Naïve rats were anesthetized with 70% carbon dioxide in air, followed by decapitation. A block of tissue that contained the entorhinal, perirhinal, and piriform cortices and amygdala was dissected as previously described^43^, homogenized with lysis buffer (50 mM KCl, 50 mM PIPES, 10 mM EGTA, 2 mM MgCl_2_, 0.5% Triton X-100, 100 μM PMSF, 1 mM DTT, and proteinase inhibitor cocktail) using TissueRuptor (Qiagen, Venlo, Netherlands), and incubated on ice for 20 min. Lysates were incubated with goat anti-MBD3 (sc9402, Santa Cruz Biotechnology, Dallas, TX, USA) antibody-coupled resin (26149, Thermo Fisher Scientific, Waltham, MA, USA) at 4 °C overnight. The resin was washed with lysis/wash buffer four times, and immunoprecipitates were eluted with elution buffer according to the manufacturer’s instructions. Three independent immunoprecipitation replicates were performed.

Mass spectrometry was performed at the Mass Spectrometry Laboratory at the Institute of Biochemistry and Biophysics (Warsaw, Poland) using liquid chromatography (nonoAcquity UPLC, Waters, Milford, MA, USA) and an Orbitrap Velos Pro TM Hybrid Ion Trap-Orbitrap mass spectrometer (Thermo Fisher Scientific, Waltham, MA, USA). The data were analyzed using Mascot software (http://www.matrixscience.com).

For Western blot, immunoprecipitates were subjected to Tris-glycine sodium dodecyl sulphate-polyacrylamide gel electrophoresis (SDS-PAGE) and transferred to nitrocellulose membranes Hybond-ECL (Amersham, Amersham, UK). For immunodetection, nonspecific binding was blocked by incubation in 5% non-fat milk in TBS-T (0.5 M Tris, 0.9% NaCl, 0.1% Tween 20, pH 8). The proteins were then probed with primary antibodies (goat anti-MBD3, sc9402, Santa Cruz Biotechnology, Dallas, TX, USA; rabbit anti-MTA2, sc28731, Santa Cruz Biotechnology, Dallas, TX, USA; rabbit anti-HDAC1, sc7872, Santa Cruz Biotechnology, Dallas, TX, USA; rabbit anti-HDAC2, ab16032, Abcam, Cambridge, UK; rabbit anti-GATAD2B, #HPA017015, Atlas Antibodies; rabbit anti-MTA1, #A300-280A-T, Bethyl Laboratories, Montgomery, TX, USA; rabbit anti-RbBP4, #A301-206A-T, Bethyl Laboratories, Montgomery, TX, USA), followed by secondary horseradish peroxidase-conjugated antibodies (goat anti-rabbit conjugated with horseradish peroxidase antibody, AP132P, Merck Millipore, Darmstadt, Germany; horse anti-goat conjugated with horseradish peroxidase antibody, PI-9500, Vector Laboratories, Burlingame, CA, USA, mouse anti-rabbit conjugated with horseradish peroxidase antibody, 211-032-171, Jackson ImmunoResearch Laboratories, West Grove, PA, USA). Signals were detected by chemiluminescence with ECL Prime Western Blotting Detection Reagent (RPN2232 Amersham, Amersham, UK) according to the manufacturer’s instructions. The membranes were then re-probed with other antibodies.

### Immunofluorescence

Animals were euthanized at 14 d after stimulation by intraperitoneal injections of morbital (200 mg/kg) and transcardially perfused with 4% formaldehyde in 0.1 M phosphate buffer (pH 7.4). The brains were post-fixed for 4 h, cryoprotected in 30% sucrose for 3–4 days, frozen on dry ice, and stored at −70 °C. Coronal sections (30 μm) were cut and stored in a cryoprotectant solution (30% ethylene glycol, 25% glycerol, 0.05 M phosphate buffer) at −20 °C. Immunohistochemistry was performed with free-floating coronal brain sections using a standard procedure^26^. Briefly, for goat anti-MBD3 (sc9402, Santa Cruz Biotechnology, Dallas, TX, USA), rabbit anti-MTA2 (sc28731, Santa Cruz Biotechnology, Dallas, TX, USA), rabbit anti-HDAC2 (ab16032, Abcam, Cambridge, UK), and rabbit anti-Olig2 (AB9610, Merck Millipore, Darmstadt, Germany), the sections were pretreated with a solution that contained 10 mM sodium citrate (pH 6.0) and 0.05% Tween 20 for 30 min at 80 °C for antigen retrieval. After blocking for nonspecific binding for 2 h with PBS that contained 10% horse or goat serum (Vector Laboratories, Burlingame, CA, USA) and 0.2% Triton X-100, a primary antibody (diluted in PBS that contained 1% serum and 0.2% Triton X-100) was applied, and the sections were incubated overnight at 4 °C. Subsequently, the sections were incubated for 2 h with the appropriate secondary antibody diluted in PBS (0.2% Triton X-100). The sections were then labeled with antibodies against mouse anti-NeuN (MAB377, Merck Millipore, Darmstadt, Germany), mouse anti-GFAP-Cy3 (C9205, Sigma-Aldrich, St. Louis, MO, USA), mouse anti-CD11b (MCA275G, AbD Serotec, Puchheim, Germany), or mouse anti-APC-CC1 (OP80, Calbiochem, San Diego, CA, USA). The nuclei were counterstained with 4,6-diamidino-2-phenylindole (DAPI; Sigma Aldrich, St. Louis, MO, USA). The sections were than mounted on gelatin-coated slides and coverslipped in Vectashield (H1000, Vector Laboratories, Burlingame, CA, USA). To visualize MBD3, MTA2, and HDAC2 co-localization with cellular markers, the sections were examined under a confocal laser-scanning microscope (LSM780, Carl Zeiss, Oberkochen, Germany). Three-dimensional reconstructions were performed with Imaris software (Bitplane, Zurich, Switzerland) followed by deconvolution using AutoQuant X2 (Media Cybernetics, Rockville, MD, USA). The results were confirmed in three independent brain sections from both sham-operated (*n* = 5) and stimulated (*n* = 5) animals. All stimulated animals had spontaneous seizures (Table 2).

### Quantification of intensity of MBD3 immunoreactivity

All of the quantifications were performed using coronal brain sections that spanned rostrocaudal levels −2.04 mm anterior to bregma to −7.92 mm posterior to bregma and were stained for MBD3 and neuronal marker NeuN. All sections containing structures of interest were selected. Twelve to 15 sections from 1-in-5 series from each brain were analyzed. For calculations of the immunofluorescence intensity of MBD3 protein in neurons and the number of positively stained cells for MBD3 and NeuN antibodies, images of brain sections were collected under a fluorescent microscope (Eclipse 80i, Nikon, Tokyo, Japan) using ImagePro Plus 5.0 software (Media Cybernetics, Rockville, MD, USA). Borders of the entorhinal and piriform cortices and amygdala nuclei were manually drawn on each brain section using Photoshop CS5 software (Adobe, San Jose, CA, USA) with guidance from a rat brain atlas^44^ and NeuN staining. Images of the stained sections were analyzed using ImageJ 1.46r software (National Institutes of Health, Bethesda, MD, USA). Briefly, for each image field, the total number of pixels was quantified on a gray scale (0–255). The levels of immunoreactivity are expressed as the average density (mean pixel value in an image field that covered corresponding structures ± SD) for each section. To examine the percentage of neurons that contained MBD3, the masks, including NeuN-positive cells in each structure, were used to identify double-labeled cells that expressed both MBD3 and NeuN. Statistical analyses were performed using Prism 5.0 software (GraphPad, La Jolla, CA, USA). All of the data are expressed as the mean ± standard deviation (SD; *n* = 5). All stimulated animals had spontaneous seizures (Table 2). Differences in parameters were analyzed using two-way analysis of variance followed by Tukey post hoc test.

### ChIP-Seq and ChIP-qPCR

For ChIP-Seq and ChIP-quantitative polymerase chain reaction (qPCR) assays, blocks of tissue that contained entorhinal, perirhinal, and piriform cortices and the amygdala, dissected as described previously^43^, were sliced into 1 mm pieces and crosslinked with a 1% formaldehyde solution for 15 min at room temperature. Fixation was quenched by 125 mM glycine. The tissue was washed twice with ice-cold PBS and stored at −80 °C. Samples were thawed on ice, resuspended in 1 ml of PBS that contained protease inhibitor cocktail (11697498001, Roche Diagnostics, Basel, Switzerland) and 1 mM PMSF, and homogenized using TissueRuptor (Qiagen, Venlo, Netherlands). The homogenates were centrifuged at 2300 × *g* for 5 min at 4 °C. The supernatant was removed, and the pellet was resuspended in 1.8 ml of lysis buffer (50 mM Tris-HCl, 10 mM ethylenediaminetetraacetic acid, and 1% SDS, pH 8.1) that contained protease inhibitor cocktail. After 1-h incubation at 4 °C with rotation, cells were physically ground in an ice-cold Dounce homogenizer (Wheaton, NJ, USA) with 30 strokes to release nuclei. Samples were sonicated with Bioruptor Plus UCD-300 (Diagenode, Seraing, Belgium) for 105 min in seven cycles of 30 s on and off at the high setting. Sheared chromatin was centrifuged at 12000 × *g* for 15 min at 4 °C, and the supernatant was collected and stored at −80 °C. Next, 420 μl of the supernatant (25 μg chromatin) was diluted 10 times in ChIP Buffer 1 and incubated with 26 μl of protein G magnetic beads and 4 μg anti-MBD3 antibody (A302-528A, Bethyl Laboratories, Montgomery, TX, USA) at 4 °C overnight with rotation according to the manufacturer’s instructions (53008, Active Motif, Carlsbad, CA, USA). The beads were washed once with 800 μl of ChIP Buffer 1 and twice with 800 μl of ChIP Buffer 2 and resuspended with 50 μl of Elution Buffer AM2. The samples were incubated for 15 min at room temperature on an end-to-end rotator, and 50 μl of Reverse Cross-linking Buffer was added to the eluted chromatin. Eluted DNA was incubated at 65 °C overnight, followed by incubation for 30 min at 37 °C with 1 μl of RNase A (Thermo Fisher Scientific, Waltham, MA, USA). The samples were then treated with proteinase K (03 115 887 001, Roche Diagnostics, Basel, Switzerland) for 1.5 h at 42 °C. DNA was then purified by phenol-chloroform extraction and ethanol precipitation. ChIP-Seq libraries were prepared using custom inline bar-coded adaptors (NEBNext Multiplex Oligos for Illumina, New England BioLabs, Ipswich, MA, USA) and sequenced on the Illumina HiSeq2000 platform at the European Molecular Biology Laboratory (Heidelberg, Germany). Data from n = 3 sham operated rats and n = 4 stimulated rats passed stringent quality criteria (see ChIP-Seq analysis section) and only those animals were enrolled in the analysis. Spontaneous seizures were detected in two stimulated animals (Table 2).

ChIP-qPCR was performed using SYBR Green Real-Time PCR Master Mix (Applied Biosystems, Foster City, CA, USA). The data were evaluated using ABI PRISM 7500 Sequence Detection and corresponding software (Applied Biosystems, Foster City, CA, USA). The data were normalized to input samples for the amount of chromatin and immunoprecipitation efficiency using normal IgG controls. Average C_t_ values for individual ChIP and IgG controls are expressed as a percentage of input. Data were obtained from sham operated and stimulated rats (n = 5). Spontaneous seizures were detected in two animals (Table 2). Quantitative PCR was performed in triplicate using the following primers:

*Pop1* forward: AGTCAGGGACCCGAGAGTAAA

*Pop1* reverse: CACTCACCGTGGTATCGTTCT

*Syt8* forward: CCACTGCCCTCTCAAGGATG

*Syt8* reverse: CTGCTCCTCTGCCAATCCTG

*Cyp2c6v1* forward: TCTGCAATGTGAAGAGAATGAAACC

*Cyp2c6v1* reverse: TTAGCAGTCTCAAGGCAAGTGT

*Tacc2* forward: ACAAGCTCATGCCGGGTTACT

*Tacc2* reverse: CAACTGACACTGGCTTGGATCTGA

*Nfia* forward: AAAGGCCTGTCCGTTCTCCT

*Nfia* reverse: AACCTCTGTCTGCACCGTGA

*Ddx60* forward: ACAAGAAAGGAGCCAGAGGA

*Ddx60* reverse: CAGAGGATTGTTGTTCCAGGG

*Klhl14* forward: AAGGTCTCCTTCGTTACGGC

*Klhl14* reverse: TTCATCCCCTGAACCAAAGTAGG

*Rbms3* forward: ACTTGCTTCTAGCAGAGTTCTT

*Rbms3* reverse: TTCCTGACAAAAGTGTCATCATATC

*Irg1* forward: TGTCTTTTTGATTCCTTCTGTCGT

*Irg1* reverse: TCTGAATAAGCACCTGTCGATT

### ChIP-Seq analysis

Biological replicates were processed individually. The fastq files were filtered with fastq_quality_filter (FASTX-Toolkit version 0.0.14)^45^ to obtain reads for which 90% of the sequence was sequenced with a sequencing error ≤1%. Adapter contamination was removed with cutadapt 1.7.1^46^. Subsequently, reads were mapped to the rat genome version RN5 using Bowtie 1.0.0^47^ with options -a -v 3 -m 1 --best --strata.

MBD3 peaks were detected with MACS2 2.1.0.20141030^48^ with a fixed extension size of 150 bp and a significance cut-off at FDR equal to 1e-1 and effective genome size of 1.87e9 and post-processing option --call-summits. Each sham sample was tested against the common for sham samples input DNA obtained from one of the sham animals, and each stimulated sample was tested against the common for stimulated samples input DNA obtained from one of the stimulated animals.

Peak detection was followed by differential binding analysis with R/Bioconductor package DiffBind 1.12.1 under R 3.1.2 software^49^. Counts were normalized with Trimmed Mean of M-values (TMM) obtained with edgeR using ChIP read counts minus control read counts and full library size. MBD3 binding sites that were detected in at least two samples were tested with the edgeR algorithm for differential binding between amygdala-stimulated and sham-operated animals. Sites with *P* < 0.01 were considered differentially occupied by MBD3 protein. The data are expressed as a heatmap that represents relative (z-score) differences in the binding of MBD3 protein between sham-operated and amygdala-stimulated animals (cut-off FDR < 0.1).

ChIP-Seq peaks were defined as associated with genomic features if they intersected at least 1 bp. Information about genomic features was obtained from the Ensembl 79 (RN5) database via the UCSC Genome Browser^50^. The genomic features that were queried included enhancers (from 2 to 10 kb upstream from transcriptional start site [TSS]), promoters (500 bp downstream and 2 kb upstream from TSS), TSS, 5′UTR (5′ untranslated region), the coding part of the exons, introns, gene bodies, 3′UTR (3′ untranslated region), transcriptional end site (TES), 2 kb downstream from TES, and CpG islands.

The overrepresentation of GO Biological Process terms that were enriched in genes in which the differential binding of MBD3 protein in gene bodies that were found was analyzed using the gProfileR webservice^51^.

The distribution of sites that were differentially (cut-off *P* < 0.01) occupied by MBD3 at enhancers (from 2 to 10 kb upstream from TSS), promoters (500 bp downstream and 2 kb upstream from TSS), TSS, 5′UTR, the coding part of the exons, introns, gene bodies (TSS to TES), 3′UTR, TES, 2 kb downstream from TES, non-genic regions, and CpG islands was analyzed. The data are expressed as the percent (%) of MBD3 binding sites associated with genomic features. The frequency of observed sites that were differentially occupied by MBD3 compared with non-differentially occupied by MBD3 with upper and lower confidence intervals for different genomic features was also examined. The significance of differences in frequencies was estimated using Fisher’s Exact test (*P < 0.05, **P < 0.01, ***P < 0.001).

### RNA isolation and qRT-PCR

Total RNA was extracted from the temporal lobe structures using RNeasy Mini kit according to the manufacturer’s instructions (Qiagen, Venlo, Netherlands). cDNAs were synthesized with the High Capacity RNA-to-cDNA Kit (Applied Biosystems, Waltham, MA, USA). The resulting cDNA was used in triplicate SYBR Green reactions (Applied Biosystems, Foster City, CA). *Hprt1* (hypoxanthine phosphoribosyltransferase 1) expression was used as a control. Fold changes relative to control were calculated and are presented as the value for each single animal and the mean ± SD. Data were obtained from sham operated animals (n = 6) and stimulated animals (n = 6). Two stimulated animals had spontaneous seizures (Table 2). For comparisons groups, two-tailed *t*-tests were performed. The following primers were used in the qRT-PCR assays:

*Astn1* forward: TCCCCGGAGAAATGGTCGTA

*Astn1* reverse: CCTGAGATCTCCAGCACGAAG

*Stxa1* forward: CCATCTTTGCCTCTGGGATCA

*Stxa1* reverse: GTGCCTGGTCTCGATCTCAC

*Ap3b2* forward: ACCGTGATGAGCTTGTGGTT

*Ap3b2* reverse: GTTTGATGATCTCGCCGTGC

*Sema3e* forward: CAGCAATTTGTGGGAGACGC

*Sema3e* reverse: TTGTAGTGATCGTGGGGTGC

*Ptprt* forward: CCATCAAAAGGAGGAAGCTGGC

*Ptprt* reverse: CCTTCATCGTTGCGGTTGGT

*Lama4* forward: TCACCACACCGATGGCTAAC

*Lama4* reverse: TGAGGTTTCTCACTGCGTCC

*Hcn1* forward: CCGGATTGCTGGGTTTCTCT

*Hcn1* reverse: CGCCATAACCAATGCACAGC

*Stat2* forward: TGTTTCCCAATGGCCCTACC

*Stat2* reverse: TGGGCTGCATCTCTTGAGC

*Sp8* forward: ACTTCACTTCTAGGGGAAGAACC

*Sp8* reverse: CTGGGGCTGCCGATCTTATT

*Hprt1* forward: CTCATGGACTGATTATGGACAGGAC

*Hprt1* reverse: GCAGGTCAGCAAAGAACTTATAGCC

## Additional Information

**How to cite this article**: Bednarczyk, J. *et al.* MBD3 expression and DNA binding patterns are altered in a rat model of temporal lobe epilepsy. *Sci. Rep.* **6**, 33736; doi: 10.1038/srep33736 (2016).

## Supplementary Material

Supplementary Information

## Figures and Tables

**Table 1:** Proteins coprecipitated with MBD3 in the immunoprecipitation assay with the antibody directed against MBD3 followed by mass spectrometry.

Protein name	Selected protein functions	Peptide #	
		Anty-IgG	anty-MBD3
PPP2CB, Serine/threonine-protein phosphatase 2A catalytic subunit beta isoform	modulates the activity of phosphorylase B kinase casein kinase 2, mitogen-stimulated S6 kinase, and MAP-2 kinase	0	5
APAF1, Apoptotic protease-activating factor 1	mediates the cytochrome c-dependent autocatalytic activation of pro-caspase-9 (Apaf-3), leading to the activation of caspase-3 and apoptosis	0	5
**HDAC2, Histone deacetylase 2**	deacetylates lysine residues on the N-terminal part of the core histones (H2A, H2B, H3 and H4)	0	3
**MBD3, Methyl-CpG binding domain protein 3**	acts as transcriptional repressor and plays a role in gene silencing; binds to DNA with a preference for sites containing methylated CpG dinucleotides; recruits histone deacetylases and DNA methyltransferases	0	2
ILF2, Interleukin enhancer-binding factor 2	transcription factor regulating transcription of the IL2 gene	0	2
NEFM, Neurofilament medium polypeptide	involved in the maintenance of neuronal caliber	0	2
**GATAD2B, Transcriptional repressor p66-beta**	Enhances MBD2-mediated repression; targets MBD3 to discrete loci in the nucleus	0	1
**RBBP4, Histone-binding protein RBBP4**	core histone-binding subunit that may target chromatin assembly factors, chromatin remodeling factors and histone deacetylases to their histone substrates in a manner that is regulated by nucleosomal DNA; component of several complexes which regulate chromatin metabolism.	0	1
**MTA1/2, Metastasis-associated protein 1/2**	transcriptional coregulator which can act as both a transcriptional corepressor and coactivator.	0	1
SRSF1, Serine/arginine-rich splicing factor 1	can either activate or repress splicing, depending on its phosphorylation state and its interaction partners	0	1
AMPD3, Adenosine monophosphate deaminase 3	plays a critical role in energy metabolism.	0	1
PPP1R9A, Neurabin-1	binds to actin filaments; may be involved in neurite formation	0	1
AAK1, AP2-associated protein kinase 1	Regulates clathrin-mediated	0	1
SYT7, Synaptotagmin-7	involved in Ca^2+^-dependent exocytosis of secretory vesicles	0	1

proteins that constitute NuRD complex are indicated in bold; protein functions were assigned on the basis of http://www.uniprot.org and http://www.genecards.org.

**Table 2:** Seizure latency to the first spontaneous seizure, number of seizures, average seizure duration and time from the last seizure to tissue collection in stimulated rats used in respective experiments.

Rat#	Disease phenotype	Experiment	Latency to the first spontaneous seizure (days)	Seizure number	Seizure duration (sec)	Time from the last seizure to tissue collection
#6	non-epileptic	*In situ* hybridization	—	0	—	—
#8	epileptic	*In situ* hybridization	7	8	38 ± 10	6 d 09 h 00 min
#9	non-epileptic	*In situ* hybridization	—	0	—	—
#15	non-epileptic	*In situ* hybridization	—	0	—	—
#42	epileptic	Immunohistochemistry	3	6	51 ± 50	2 d 17 h 54 min
#49	epileptic	Immunohistochemistry	8	5	49 ± 15	1 d 12 h 55 min
#86	epileptic	Immunohistochemistry	3	23	38 ± 31	2 d 13 h 05 min
#88	epileptic	Immunohistochemistry	3	16	74 ± 46	4 d 22 h 51 min
#91	epileptic	Immunohistochemistry	3	7	77 ± 52	17 h 27 min
#4	non-epileptic	ChIP-Seq and ChIP-PCR	—	0	—	—
#5	epileptic	ChIP-Seq and ChIP-PCR	13	7	90 ± 20	6 h 11 min
#11	epileptic	ChIP-Seq and ChIP-PCR	9	4	64 ± 37	4 d 19 h 48 min
#12	non-epileptic	ChIP-Seq and ChIP-PCR	—	0	—	—
#14	non-epileptic	ChIP-Seq and ChIP-PCR	—	0	—	—
#64	non-epileptic	qRT-PCR	—	0	—	—
#66	non-epileptic	qRT-PCR	—	0	—	—
#67	non-epileptic	qRT-PCR	—	0	—	—
#70	epileptic	qRT-PCR	8	4	55 ± 4	3 d 16 h 54 min
#71	epileptic	qRT-PCR	10	5	55 ± 19	1 d 2 h 25 min
#73	non-epileptic	qRT-PCR	—	0	—	—

The numbers of sham-operated animals used for each experiments are as follws: *In situ* hybridization – n = 4; Immunohistochemistry – n = 5; ChIP-Seq and ChIP-PCR – n = 5; qRT-PCR – n = 6.

## References

[b1] Porter R. J. In A textbook of epilepsy (eds Laidlaw J. *et al.*) 1–22 (Churchil Livingstone, 1993).

[b2] Hauser W. A. In Epilepsy: a Comprehensive Textbook (eds Engel J. Jr & Pedley T. A.) 47–57 (Lippincott-Raven Publishers, 1997).

[b3] Pitkanen A. & Sutula T. P. Is epilepsy a progressive disorder? Prospects for new therapeutic approaches in temporal-lobe epilepsy. Lancet Neurol 1, 173–181 (2002). 12849486 10.1016/s1474-4422(02)00073-x

[b4] Pitkanen A. & Lukasiuk K. Mechanisms of epileptogenesis and potential treatment targets. Lancet Neurol 10, 173–186 (2011). 21256455 10.1016/S1474-4422(10)70310-0

[b5] Pitkänen A., Lukasiuk K., Dudek F. E. & Staley K. J. Epileptogenesis. Cold Spring Harb Perspect Med 5, doi: 10.1101/cshperspect.a022822 (2015). PMC4588129 26385090

[b6] Qureshi I. A. & Mehler M. F. Epigenetic mechanisms underlying human epileptic disorders and the process of epileptogenesis. Neurobiol Dis 39, 53–60, doi: 10.1016/j.nbd.2010.02.005 (2010). 20188170 PMC2874104

[b7] Kobow K. & Blümcke I. Epigenetic mechanisms in epilepsy. Prog Brain Res 213, 279–316, doi: 10.1016/B978-0-444-63326-2.00014-4 (2014). 25194494

[b8] Ryley Parrish R. *et al.* Status epilepticus triggers early and late alterations in brain-derived neurotrophic factor and NMDA glutamate receptor Grin2b DNA methylation levels in the hippocampus. Neuroscience 248, 602–619, doi: 10.1016/j.neuroscience.2013.06.029 (2013). 23811393 PMC3830613

[b9] Williams-Karnesky R. L. *et al.* Epigenetic changes induced by adenosine augmentation therapy prevent epileptogenesis. J Clin Invest 123, 3552–3563, doi: 10.1172/JCI65636 (2013). 23863710 PMC3726154

[b10] Zhu Q. *et al.* Increased expression of DNA methyltransferase 1 and 3a in human temporal lobe epilepsy. J Mol Neurosci 46, 420–426, doi: 10.1007/s12031-011-9602-7 (2012). 21826395

[b11] Miller-Delaney S. F. *et al.* Differential DNA methylation profiles of coding and non-coding genes define hippocampal sclerosis in human temporal lobe epilepsy. Brain 138, 616–631, doi: 10.1093/brain/awu373 (2015). 25552301 PMC4408428

[b12] Kobow K. *et al.* Deep sequencing reveals increased DNA methylation in chronic rat epilepsy. Acta Neuropathol 126, 741–756, doi: 10.1007/s00401-013-1168-8 (2013). 24005891 PMC3825532

[b13] Kobow K. *et al.* Increased reelin promoter methylation is associated with granule cell dispersion in human temporal lobe epilepsy. J Neuropathol Exp Neurol 68, 356–364, doi: 10.1097/NEN.0b013e31819ba737 (2009). 19287316

[b14] Machnes Z. M. *et al.* DNA methylation mediates persistent epileptiform activity *in vitro* and *in vivo*. Plos One 8, e76299, doi: 10.1371/journal.pone.0076299 (2013). 24098468 PMC3788713

[b15] Du Q., Luu P. L., Stirzaker C. & Clark S. J. Methyl-CpG-binding domain proteins: readers of the epigenome. Epigenomics 1–23, doi: 10.2217/epi.15.39 (2015). 25927341

[b16] Nissenkorn A. *et al.* Epilepsy in Rett syndrome—the experience of a National Rett Center. Epilepsia 51, 1252–1258, doi: 10.1111/j.1528-1167.2010.02597.x (2010). 20491871

[b17] Della Sala G. & Pizzorusso T. Synaptic plasticity and signaling in Rett syndrome. Dev Neurobiol 74, 178–196, doi: 10.1002/dneu.22114 (2014). 23908158

[b18] Lukasiuk K., Kontula L. & Pitkanen A. cDNA profiling of epileptogenesis in the rat brain. Eur J Neurosci 17, 271–279 (2003). 12542663 10.1046/j.1460-9568.2003.02461.x

[b19] Yamada T. *et al.* Promoter decommissioning by the NuRD chromatin remodeling complex triggers synaptic connectivity in the mammalian brain. Neuron 83, 122–134, doi: 10.1016/j.neuron.2014.05.039 (2014). 24991957 PMC4266462

[b20] Knock E. *et al.* The methyl binding domain 3/nucleosome remodelling and deacetylase complex regulates neural cell fate determination and terminal differentiation in the cerebral cortex. Neural Dev 10, 13, doi: 10.1186/s13064-015-0040-z (2015). 25934499 PMC4432814

[b21] Cukier H. N. *et al.* Novel variants identified in methyl-CpG-binding domain genes in autistic individuals. Neurogenetics 11, 291–303, doi: 10.1007/s10048-009-0228-7 (2010). 19921286 PMC2941261

[b22] Guzik-Kornacka A., Sliwa A., Plucinska G. & Lukasiuk K. Status epilepticus evokes prolonged increase in the expression of CCL3 and CCL4 mRNA and protein in the rat brain. Acta Neurobiol Exp (Wars) 71, 193–207 (2011). 21731074 10.55782/ane-2011-1840

[b23] Nissinen J., Halonen T., Koivisto E. & Pitkanen A. A new model of chronic temporal lobe epilepsy induced by electrical stimulation of the amygdala in rat. Epilepsy Res 38, 177–205 (2000). 10642046 10.1016/s0920-1211(99)00088-1

[b24] Tuunanen J., Lukasiuk K., Halonen T. & Pitkanen A. Status epilepticus-induced neuronal damage in the rat amygdaloid complex: distribution, time-course and mechanisms. Neuroscience 94, 473–495 (1999). 10579210 10.1016/s0306-4522(99)00251-1

[b25] Jung B. P. *et al.* Differential expression of methyl CpG-binding domain containing factor MBD3 in the developing and adult rat brain. J Neurobiol 55, 220–232, doi: 10.1002/neu.10199 (2003). 12672019

[b26] Stefaniuk M. & Lukasiuk K. Cloning of expressed sequence tags (ESTs) representing putative epileptogenesis-related genes and the localization of their expression in the normal brain. Neurosci Lett 482, 230–234 (2010). 20655365 10.1016/j.neulet.2010.07.045

[b27] Lee C. *et al.* Members of the NuRD chromatin remodeling complex interact with AUF1 in developing cortical neurons. Cereb Cortex 18, 2909–2919, doi: 10.1093/cercor/bhn051 (2008). 18413351 PMC2724833

[b28] MacDonald J. L. & Roskams A. J. Histone deacetylases 1 and 2 are expressed at distinct stages of neuro-glial development. Dev Dyn 237, 2256–2267, doi: 10.1002/dvdy.21626 (2008). 18651664

[b29] Yao Z. G. *et al.* Regional and cell-type specific distribution of HDAC2 in the adult mouse brain. Brain Struct Funct 218, 563–573, doi: 10.1007/s00429-012-0416-3 (2013). 22532304

[b30] Yoo J. Y., Larouche M. & Goldowitz D. The expression of HDAC1 and HDAC2 during cerebellar cortical development. Cerebellum 12, 534–546, doi: 10.1007/s12311-013-0459-x (2013). 23436026

[b31] Ballas N., Lioy D. T., Grunseich C. & Mandel G. Non-cell autonomous influence of MeCP2-deficient glia on neuronal dendritic morphology. Nat Neurosci 12, 311–317, doi: 10.1038/nn.2275 (2009). 19234456 PMC3134296

[b32] Nguyen M. V. *et al.* Oligodendrocyte lineage cells contribute unique features to Rett syndrome neuropathology. J Neurosci 33, 18764–18774, doi: 10.1523/JNEUROSCI.2657-13.2013 (2013). 24285883 PMC3841446

[b33] Maezawa I., Swanberg S., Harvey D., LaSalle J. M. & Jin L. W. Rett syndrome astrocytes are abnormal and spread MeCP2 deficiency through gap junctions. J Neurosci 29, 5051–5061, doi: 10.1523/JNEUROSCI.0324-09.2009 (2009). 19386901 PMC3436907

[b34] Schultz D. C., Friedman J. R. & Rauscher F. J. Targeting histone deacetylase complexes via KRAB-zinc finger proteins: the PHD and bromodomains of KAP-1 form a cooperative unit that recruits a novel isoform of the Mi-2alpha subunit of NuRD. Genes Dev 15, 428–443, doi: 10.1101/gad.869501 (2001). 11230151 PMC312636

[b35] Francis J. *et al.* Kindling induces the mRNA expression of methyl DNA-binding factors in the adult rat hippocampus. Neuroscience 113, 79–87 (2002). 12123686 10.1016/s0306-4522(02)00150-1

[b36] Jung B. P., Zhang G., Ho W., Francis J. & Eubanks J. H. Transient forebrain ischemia alters the mRNA expression of methyl DNA-binding factors in the adult rat hippocampus. Neuroscience 115, 515–524 (2002). 12421618 10.1016/s0306-4522(02)00383-4

[b37] Günther K. *et al.* Differential roles for MBD2 and MBD3 at methylated CpG islands, active promoters and binding to exon sequences. Nucleic Acids Res 41, 3010–3021, doi: 10.1093/nar/gkt035 (2013). 23361464 PMC3597697

[b38] Shimbo T. *et al.* MBD3 localizes at promoters, gene bodies and enhancers of active genes. PLoS Genet 9, e1004028, doi: 10.1371/journal.pgen.1004028 (2013). 24385926 PMC3873231

[b39] Saitsu H. *et al.* De novo mutations in the gene encoding STXBP1 (MUNC18-1) cause early infantile epileptic encephalopathy. Nat Genet 40, 782–788, doi: 10.1038/ng.150 (2008). 18469812

[b40] Lim S. H., Moon J., Lee M. & Lee J. R. PTPRT regulates the interaction of Syntaxin-binding protein 1 with Syntaxin 1 through dephosphorylation of specific tyrosine residue. Biochem Biophys Res Commun 439, 40–46, doi: 10.1016/j.bbrc.2013.08.033 (2013). 23962429

[b41] Powell K. L. *et al.* Decreases in HCN mRNA expression in the hippocampus after kindling and status epilepticus in adult rats. Epilepsia 49, 1686–1695, doi: 10.1111/j.1528-1167.2008.01593.x (2008). 18397293

[b42] Winawer M. R. *et al.* A locus on mouse Ch10 influences susceptibility to limbic seizure severity: fine mapping and in silico candidate gene analysis. Genes Brain Behav 13, 341–349, doi: 10.1111/gbb.12116 (2014). 24373497 PMC3947448

[b43] Nikolaev E., Kaminska B., Tischmeyer W., Matthies H. & Kaczmarek L. Induction of expression of genes encoding transcription factors in the rat brain elicited by behavioral training. Brain Res Bull 28, 479–484 (1992). 1375525 10.1016/0361-9230(92)90050-8

[b44] Paxinos G. & Watson C. The Rat Brain in Stereotaxic Coordinates. sixth edn, (Elsevier, 2007). 10.1016/0165-0270(80)90021-7 6110810

[b45] Hannon-Lab *FASTX Toolkit* (2010). Available at: http://hannonlab.cshl.edu/fastx_toolkit/index.html (Accessed: 5th January 2014).

[b46] Martin M. Cutadapt removes adapter sequences from high-throughput sequencing reads. EMBnet.journal 17, 10–20 (2011).

[b47] Langmead B., Trapnell C., Pop M. & Salzberg S. L. Ultrafast and memory-efficient alignment of short DNA sequences to the human genome. Genome Biol 10, R25, doi: 10.1186/gb-2009-10-3-r25 (2009). 19261174 PMC2690996

[b48] Zhang Y. *et al.* Model-based analysis of ChIP-Seq (MACS). Genome Biol 9, R137, doi: 10.1186/gb-2008-9-9-r137 (2008). 18798982 PMC2592715

[b49] Stark R. & Brown G. D. DiffBind: diffferential binding analysis of ChIP-seq peak data (2015). Available at: http://bioconductor.org/packages/release/bioc/html/DiffBind.html (Accessed: 9th December 2014).

[b50] Kent W. J., Sugnet C. W., Furey T. S. *et al.* The human genome browser at UCSC. Genome Research 12, 996–1006 (2002). 12045153 10.1101/gr.229102 PMC186604

[b51] Reimand J., Arak T. & Vilo J. g:Profiler—a web server for functional interpretation of gene lists (2011 update). Nucleic Acids Res 39, W307–W315, doi: 10.1093/nar/gkr378 (2011). 21646343 PMC3125778

