# Supplementary material for: MBD3 expression and DNA binding patterns are altered in a rat model of temporal lobe epilepsy
Source: Sci Rep. 2016 Sep 21;6:33736. doi: 10.1038/srep33736 (PMC5030630; doi:10.1038/srep33736)
Supplement: Supplementary Information [file srep33736-s1.pdf]

SUPPLEMENTARY INFORMATION

MBD3 expression and DNA binding patterns are altered in a rat model of temporal lobe epilepsy

Joanna Bednarczyk,[1] Konrad J. Dębski,[1, 2] Anna M. Bot,[1] and Katarzyna Lukasiuk[1*]

[1] Laboratory of Epileptogenesis, Department of Molecular and Cellular Neurobiology, Nencki Institute of Experimental Biology of Polish Academy of Sciences, Warsaw, Poland

[2] Laboratory of Bioinformatics, Neurobiology Center, Nencki Institute of Experimental Biology of Polish Academy of Sciences, Warsaw, Poland

Supplementary Table 1. Percentage of neurons expressing MBD3 protein in temporal lobe structures in normal and epileptic animals.

	**SHAM**		**SE**	
	**Ipsilateral**	**Contralateral**	**Ipsilateral**	**Contralateral**
Piriform cortex (layer III)	98.4 ± 1.1%	99.9 ± 2.3%	96.8 ± 0.1%	99.4 ± 1.1%
Entorhinal cortex (layers III-VI)	99.6 ± 0.5%	99.9 ± 0.7%	99.4 ± 0.1%	99.9 ± 0.2%
Lateral nucleus of the amygdala	98.0 ± 1.5%	99.9 ± 0.4%	99.4 ± 0.1%	99.9 ± 0.1%
Central nucleus of the amygdala	99.9 ± 0.1%	99.8 ± 1.5%	99.2 ± 0.4%	99.9 ± 0.2%
Basal nucleus of the amygdala	99.7 ± 0.2%	99.9 ± 0.4%	99.5 ± 0.0%	99.8 ± 0.3%
Medial nucleus of the amygdala	99.7 ± 0.5%	99.8 ± 2.0%	98.6 ± 0.5%	99.9 ± 0.2%
Basomedial nucleus of the amygdala	99.3 ± 0.9%	99.9 ± 0.2%	99.6 ± 0.1%	99.9 ± 0.1%

Supplementary Figure 1. Full length scans of Western Blots used for images cropped to Figure 6.

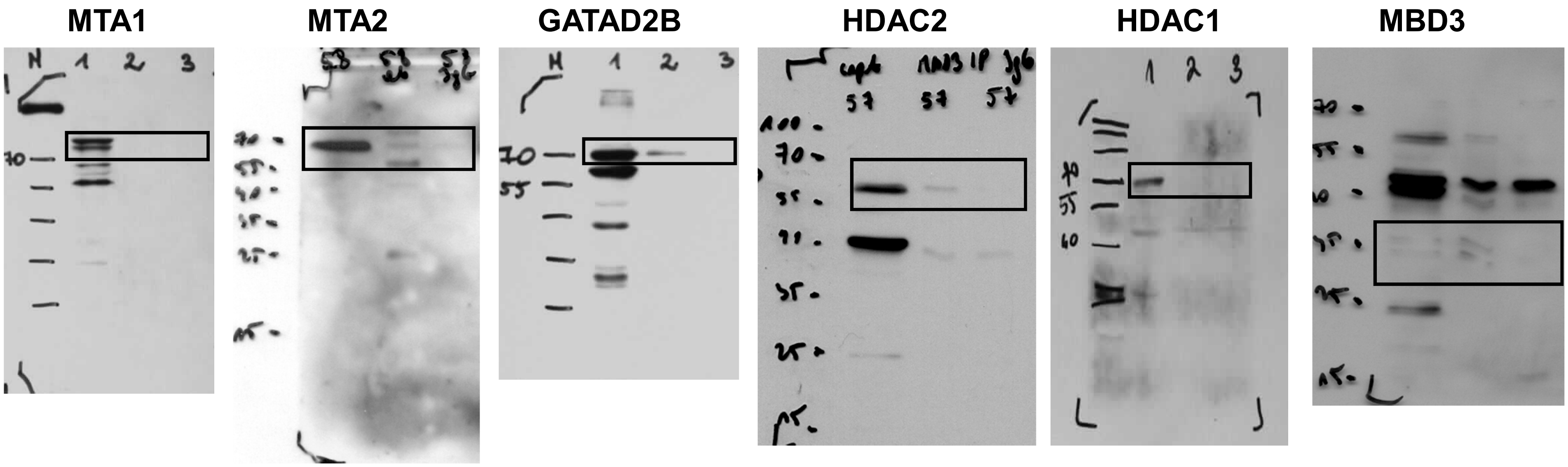

1 – input
2 – MBD3 CoiP
3 – IgG
